# Supplementary material for: Socioeconomic risk factors and human immunosuppressive conditions are major drivers of human leishmaniasis in southern Europe
Source: PLoS Negl Trop Dis. 2026 Oct 5;20(10):e0014787. doi: 10.1371/journal.pntd.0014787 (PMC13649131; doi:10.1371/journal.pntd.0014787)
Supplement: Supplementary file 1 [file pntd.0014787.s003.docx]

**Supplementary Material 3. Model formulation**

This document was constructed based on Moraga (2019, 2014) ^1,2^ and Zuur & Ieno (2017) ^3^.

When expected incidence could be calculated given data, let $Y_{i}$ and $E_{i}$ be the observed and expected number of leishmaniasis, respectively, and let $\theta_{i}$ be the relative risk for NUTS3 $i=1, \ldots., n.$ The model is specified as follows:

$Y_{i}|\theta_{i} \sim Poisson \left( E_{i} \times\theta_{i} \right), i=1, \ldots., n,$	(1)

$\log(\theta_{i})= \beta_{0}+ u_{i}+ \varepsilon$	(2)

$\log(\theta_{i})= \beta_{0}+ S\left( x_{i} \right)+ \varepsilon$	(3)

$\log\left( \theta_{i} \right)= \beta_{0}+ \beta_{1} \times{var}_{i} + \varepsilon$	(4)

$\log\left( \theta_{i} \right)= \beta_{0}+ f\left( {var}_{i} \right)+ \varepsilon$	(5)

$\log\left( \theta_{i} \right)= \beta_{0}+ \beta_{1} \times{var}_{i}+ u_{i} + \varepsilon$	(6)

$\log\left( \theta_{i} \right)= \beta_{0}+ f\left( {var}_{i} \right)+ u_{i}+ \varepsilon$	(7)

$\log\left( \theta_{i} \right)= \beta_{0}+ \beta_{1} \times{var}_{i}+ S\left( x_{i} \right)+ \varepsilon$	(8)

$\log(\theta_{i})= \beta_{0}+ f( {var}_{i})+ S\left( x_{i} \right)+ \varepsilon$	(9)

where, $\beta_{0}$ is the intercept and $\beta_{1}$ is the coefficient of the covariate ${var}_{i}$. $f\left( \cdot\right)$ is smooth function of the covariate ${var}_{i}$ follows a random walk of order 2. $u_{i}$ is a conditionally autoregressive model, $u_{i}|\mathbf{u}_{-i}\sim N(ū_{\delta_{i}}\frac{1}{\tau_{u}n_{\delta_{i}}})$. $S(\cdot)$ is a spatial random effect that follows a zero-mean Gaussian process with Matérn covariance function

$\mathrm{Cov}(S\left( x_{i} \right), S\left( x_{j} \right)= \frac{\sigma^{2}}{2^{v-1}\Gamma\left( \nu\right)}\left( \kappa||x_{i}-x_{j}|| \right)^{v}K_{v}(\kappa||x_{i}-x_{j}||),$	(10)

Where $K_{v}(\cdot)$ is the modified Bessel function of second kind and order $v>0$. $v$ is the smoothness parameter, $\sigma^{2}$ denotes the variance, and $\kappa>0$ is related to the practical range $\rho=\sqrt{8v}/\kappa$ which is the distance at which the spatial correlation is close to 0.1.

Formular (2) – (9) are null model of neighbor structure (m0_nb), null model of distance-based spatial random effect (m0_spde), generalized linear model (GLM), generalized additive model (GAM), generalized linear model with neighbor structure (spGLM_nb), generalized linear model with distance-based spatial random effect (spGLM_spde), generalized additive model with neighbor structure (spGAM_nb), generalized additive model with distance-based spatial random effect (spGAM_spde).

Extending the one variable models with the second variable and the interaction between the two variables, formula (4) – (9) become:

$\log\left( \theta_{i} \right)= \beta_{0}+ \beta_{1} \times{var}_{1,i}+ \beta_{2} \times{var}_{2,i} + \beta_{3} \times{var}_{1,i} \times{var}_{2,i}+ \varepsilon$	(11)

$\log\left( \theta_{i} \right)= \beta_{0}+ f\left( {var}_{1, i} \right)+ f\left( {var}_{2, i} \right)+ f\left( {var}_{1, i} \times{var}_{2, i} \right)+ \varepsilon$	(12)

$\log\left( \theta_{i} \right)= \beta_{0}+ \beta_{1} \times{var}_{1,i}+ \beta_{2} \times{var}_{2,i} + \beta_{3} \times{var}_{1,i} \times{var}_{2,i}{+ u}_{i} + \varepsilon$	(13)

$\log\left( \theta_{i} \right)= \beta_{0}+ f\left( {var}_{1,i} \right) + f\left( {var}_{2, i} \right)+ f\left( {var}_{1, i} \times{var}_{2, i} \right)+ u_{i}+ \varepsilon$	(14)

$\log\left( \theta_{i} \right)= \beta_{0}+ \beta_{1} \times{var}_{1,i}+\beta_{2} \times{var}_{2,i} + \beta_{3} \times{var}_{1,i} \times{var}_{2,i}+S\left( x_{i} \right)+ \varepsilon$	(15)

$\log(\theta_{i})= \beta_{0}+ f\left( {var}_{1,i} \right)+ f\left( {var}_{2, i} \right)+ f\left( {var}_{1, i} \times{var}_{2, i} \right)+ S\left( x_{i} \right)+ \varepsilon$	(16)

Where and $\beta_{2}$ is the coefficient of the covariate ${var}_{2,i}$, and $\beta_{3}$ is the coefficient of the interaction term between covariates ${var}_{1,i}$ and ${var}_{2,i}$.

When expected incidence could not be calculated given data, formula (1) - (9) and (11)-(16) become:

$Y_{i} \sim Poisson \left( E_{i} \right), i=1, \ldots., n,$	(17)

$\log(E_{I})= \beta_{0}+ u_{i}+ \varepsilon$	(18)

$\log(E_{i})= \beta_{0}+ S\left( x_{i} \right)+ \varepsilon$	(19)

$\log\left( E_{i} \right)= \beta_{0}+ \beta_{i} \times{var}_{i} + \varepsilon$	(20)

$\log\left( E_{i} \right)= \beta_{0}+ f\left( {var}_{i} \right)+ \varepsilon$	(21)

$\log\left( E_{i} \right)= \beta_{0}+ \beta_{i} \times{var}_{i}+ u_{i} + \varepsilon$	(22)

$\log\left( E_{i} \right)= \beta_{0}+ f\left( {var}_{i} \right)+ u_{i}+ \varepsilon$	(23)

$\log\left( E_{i} \right)= \beta_{0}+ \beta_{i} \times{var}_{i}+ S\left( x_{i} \right)+ \varepsilon$	(24)

$\log(E_{i})= \beta_{0}+ f( {var}_{i})+ S\left( x_{i} \right)+ \varepsilon$	(25)

$\log\left( E_{i} \right)= \beta_{0}+ \beta_{1} \times{var}_{1,i}+ \beta_{2} \times{var}_{2,i} + \beta_{3} \times{var}_{1,i} \times{var}_{2,i}+ \varepsilon$	(26)

$\log\left( E_{i} \right)= \beta_{0}+ f\left( {var}_{1, i} \right)+ f\left( {var}_{2, i} \right)+ f\left( {var}_{1, i} \times{var}_{2, i} \right)+ \varepsilon$	(27)

$\log\left( E_{i} \right)= \beta_{0}+ \beta_{1} \times{var}_{1,i}+ \beta_{2} \times{var}_{2,i} + \beta_{3} \times{var}_{1,i} \times{var}_{2,i}{+ u}_{i} + \varepsilon$	(28)

$\log\left( E_{i} \right)= \beta_{0}+ f\left( {var}_{1,i} \right) + f\left( {var}_{2, i} \right)+ f\left( {var}_{1, i} \times{var}_{2, i} \right)+ u_{i}+ \varepsilon$	(29)

$\log\left( E_{i} \right)= \beta_{0}+ \beta_{1} \times{var}_{1,i}+\beta_{2} \times{var}_{2,i} + \beta_{3} \times{var}_{1,i} \times{var}_{2,i}+S\left( x_{i} \right)+ \varepsilon$	(30)

$\log(E_{i})= \beta_{0}+ f\left( {var}_{1,i} \right)+ f\left( {var}_{2, i} \right)+ f\left( {var}_{1, i} \times{var}_{2, i} \right)+ S\left( x_{i} \right)+ \varepsilon$	(31)

For dataset with excessive zero’s, hurdle model or zero-altered Poisson (ZAP) was employed. ZAP consists of a Bernoulli regression as classifier and a zero-truncated Poisson (ZTP) for the regions where the response variable is not zero.

$Y_{i} \sim ZAP \left( E_{i}, \pi_{i} \right), i=1, \ldots., n,$	(32)

Where $\pi$ is the probability of the presence of leishmaniasis. Formulas (18)-(31) can be applied to ZTP. The Bernoulli model is defined as follows:

$\mathrm{logit} (\pi_{i})= \gamma_{0}+ u_{i}+ \varepsilon$	(33)

$\mathrm{logit} (\pi_{i})= \gamma_{0}+ S\left( x_{i} \right)+ \varepsilon$	(34)

$\mathrm{logit} \left( \pi_{i} \right)= \gamma_{0}+ \gamma_{i} \times{var}_{i} + \varepsilon$	(35)

$\mathrm{logit} \left( \pi_{i} \right)= \gamma_{0}+ f\left( {var}_{i} \right)+ \varepsilon$	(36)

$\mathrm{logit} \left( \pi_{i} \right)= \gamma_{0}+ \gamma_{i} \times{var}_{i}+ u_{i} + \varepsilon$	(37)

$\mathrm{logit} \left( \pi_{i} \right)= \gamma_{0}+ f\left( {var}_{i} \right)+ u_{i}+ \varepsilon$	(38)

$\mathrm{logit} \left( \pi_{i} \right)= \gamma_{0}+ \gamma_{i} \times{var}_{i}+ S\left( x_{i} \right)+ \varepsilon$	(39)

$\mathrm{logit} (\pi_{i})= \gamma_{0}+ f( {var}_{i})+ S\left( x_{i} \right)+ \varepsilon$	(40)

$\mathrm{logit} \left( \pi_{i} \right)= \gamma_{0}+ \gamma_{1} \times{var}_{1,i}+ \gamma_{2} \times{var}_{2,i} + \gamma_{3} \times{var}_{1,i} \times{var}_{2,i}+ \varepsilon$	(41)

$\mathrm{logit} \left( \pi_{i} \right)= \gamma_{0}+ f\left( {var}_{1, i} \right)+ f\left( {var}_{2, i} \right)+ f\left( {var}_{1, i} \times{var}_{2, i} \right)+ \varepsilon$	(42)

$\mathrm{logit} \left( \pi_{i} \right)= \gamma_{0}+ \gamma_{1} \times{var}_{1,i}+ \gamma_{2} \times{var}_{2,i} + \gamma_{3} \times{var}_{1,i} \times{var}_{2,i}{+ u}_{i} + \varepsilon$	(43)

$\mathrm{logit} \left( \pi_{i} \right)= \gamma_{0}+ f\left( {var}_{1,i} \right) + f\left( {var}_{2, i} \right)+ f\left( {var}_{1, i} \times{var}_{2, i} \right)+ u_{i}+ \varepsilon$	(44)

$\mathrm{logit} \left( \pi_{i} \right)= \gamma_{0}+ \gamma_{1} \times{var}_{1,i}+\gamma_{2} \times{var}_{2,i} + \gamma_{3} \times{var}_{1,i} \times{var}_{2,i}+S\left( x_{i} \right)+ \varepsilon$	(45)

$\mathrm{logit} (\pi_{i})= \gamma_{0}+ f\left( {var}_{1,i} \right)+ f\left( {var}_{2, i} \right)+ f\left( {var}_{1, i} \times{var}_{2, i} \right)+ S\left( x_{i} \right)+ \varepsilon$	(46)

where, $\gamma_{0}$ is the intercept and $\gamma_{1}$ is the coefficient of the covariate ${var}_{i}$ and ${var}_{1,i}$,  $\gamma_{2}$ is the coefficient of the covariate ${var}_{2,i}$, and $\gamma_{3}$ is the coefficient of the interaction term between covariates ${var}_{1,i}$ and ${var}_{2,i}$. The covariates ${var}_{i}$, ${var}_{1,i}$ and ${var}_{2,i}$ in the Bernoulli model and in ZTP can differ.

When the number of test is fixed, conditional on the true prevalence $P\left( x_{i} \right)$ at location $x_{i}$, $i=1, \ldots., n$, the number of positive results $Y_{i}$ out of $N_{i}$ people sampled follows a binomial distribution:

$Y_{i}| P\left( x_{i} \right) \sim Binomial \left( N_{i}, P\left( x_{i} \right) \right), i=1, \ldots., n,$	(47)

$\mathrm{logit} (P\left( x_{i} \right))= \beta_{0}+ u_{i}+ \varepsilon$	(48)

$\mathrm{logit} (P\left( x_{i} \right))= \beta_{0}+ S\left( x_{i} \right)+ \varepsilon$	(49)

$\mathrm{logit} \left( P\left( x_{i} \right) \right)= \beta_{0}+ \beta_{i} \times{var}_{i} + \varepsilon$	(50)

$\mathrm{logit} \left( P\left( x_{i} \right) \right)= \beta_{0}+ f\left( {var}_{i} \right)+ \varepsilon$	(51)

$\mathrm{logit} \left( P\left( x_{i} \right) \right)= \beta_{0}+ \beta_{i} \times{var}_{i}+ u_{i} + \varepsilon$	(52)

$\mathrm{logit} \left( P\left( x_{i} \right) \right)= \beta_{0}+ f\left( {var}_{i} \right)+ u_{i}+ \varepsilon$	(53)

$\mathrm{logit} \left( P\left( x_{i} \right) \right)= \beta_{0}+ \beta_{i} \times{var}_{i}+ S\left( x_{i} \right)+ \varepsilon$	(54)

$\mathrm{logit} (P\left( x_{i} \right))= \beta_{0}+ f( {var}_{i})+ S\left( x_{i} \right)+ \varepsilon$	(55)

$\mathrm{logit} (P\left( x_{i} \right))= \beta_{0}+ \beta_{1} \times{var}_{1,i}+ \beta_{2} \times{var}_{2,i} + \beta_{3} \times{var}_{1,i} \times{var}_{2,i}+ \varepsilon$	(56)

$\mathrm{logit} \left( P\left( x_{i} \right) \right)= \beta_{0}+ f\left( {var}_{1, i} \right)+ f\left( {var}_{2, i} \right)+ f\left( {var}_{1, i} \times{var}_{2, i} \right)+ \varepsilon$	(57)

$\mathrm{logit} \left( P\left( x_{i} \right) \right)= \beta_{0}+ \beta_{1} \times{var}_{1,i}+ \beta_{2} \times{var}_{2,i} + \beta_{3} \times{var}_{1,i} \times{var}_{2,i}{+ u}_{i} + \varepsilon$	(58)

$\mathrm{logit} \left( P\left( x_{i} \right) \right)= \beta_{0}+ f\left( {var}_{1,i} \right) + f\left( {var}_{2, i} \right)+ f\left( {var}_{1, i} \times{var}_{2, i} \right)+ u_{i}+ \varepsilon$	(59)

$\mathrm{logit} \left( P\left( x_{i} \right) \right)= \beta_{0}+ \beta_{1} \times{var}_{1,i}+\beta_{2} \times{var}_{2,i} +$ $\beta_{3} \times{var}_{1,i} \times{var}_{2,i}+S\left( x_{i} \right)+ \varepsilon$	(60)

$\mathrm{logit} (P\left( x_{i} \right))= \beta_{0}+ f\left( {var}_{1,i} \right)+ f\left( {var}_{2, i} \right)+ f\left( {var}_{1, i} \times{var}_{2, i} \right)+ S\left( x_{i} \right)+ \varepsilon$	(61)

In our study, we fitted a ZAP models for France. First, we fitted one-variable Bernoulli models and single-variable ZTP models and conducted variable selection. For Bernoulli models, a variable was selected if the WAIC of one of the six models was lower than the WAIC values of both null models by at least 2 ^4^. For the ZTP models, the same criterium was applied and it was only met by four variables: three education levels and one climatic variable. To limit the number of ZAP models, we selected the education variable with the lowest WAIC (which also had the largest effect size) and the climatic variable. We fitted ZAP models by combining one selected (of the 254) variable in Bernoulli model and one (of the two) selected variable in ZTP, for all possible combinations. Second, we fitted ZAP models with two-variable combinations in the Bernoulli part and a single variable in the ZTP part. To do that, we selected one variable per category (SM2) with the lowest WAIC and that must be lower than the WAICs of the null models by 2. ^4^ Then, each variable from those selected was paired in turn with every variable from the full set to fit six models (i.e., the three GLMs and the three GAMs) containing both variables and their interaction, except when VIF was ≥ 5 ^5^ and / or sample size was ≤ 45 (15 observations per variable / interaction).

Reference

1	Moraga P. Geospatial Health Data: Modeling and Visualization with R-INLA and Shiny. Chapman & Hall/CRC Biostatistics Series, 2019 https://www.paulamoraga.com/book-geospatial/index.html (accessed March 9, 2025).

2	Moraga Paula. Spatial statistics for data science : theory and practice with R. CRC Press, 2024.

3	Zuur AF, Ieno EN. GAM and Zero-Inflated Models. 2017.

4	Burnham KP, Anderson DR. Model Selection and Multimodel Inference: A Practical Information-Theoretic Approach, Second Edition. Fort Collins: Springer.

5	Akinwande MO, Dikko HG, Samson A, Akinwande MO, Dikko HG, Samson A. Variance Inflation Factor: As a Condition for the Inclusion of Suppressor Variable(s) in Regression Analysis. *Open J Stat* 2015; **5**: 754–67.
