# Supplementary material for: Socioeconomic risk factors and human immunosuppressive conditions are major drivers of human leishmaniasis in southern Europe
Source: PLoS Negl Trop Dis. 2026 Oct 5;20(10):e0014787. doi: 10.1371/journal.pntd.0014787 (PMC13649131; doi:10.1371/journal.pntd.0014787)
Supplement: Supplementary file 2 [file pntd.0014787.s005.docx]

Response to Reviewers

Dear editors and dear reviewer,

Thank you for the time you invested in reviewing and providing constructive feedback on our submitted manuscript. We incorporated all comments in a revised manuscript, and we outline the changes in detail below (starting with 🡪). We hope that the revised manuscript will now be suitable for publication in the journal. Thank you again for your consideration.

Best regards,

Danyang Wang

On behalf of all co-authors

**Additional Editor Comments (if provided):**

The study is very intersting and important in the field. The methodology is more complicates and possibly, a flow chart showing all the steps of analysis could be facilitate the reading of the mansucript. The discussion is excelent and hopefully, very soon we can accept the manuscript for publication.

- Thank you for your positive assessment and constructive feedback. We have now included a flowchart in the Methods section (lines 168-173) to improve the readability of the manuscript and facilitate understanding of our analysis pipeline.

**Reviewers' Comments:**

Reviewer's Responses to Questions

**Key Review Criteria Required for Acceptance?**


As you describe the new analyses required for acceptance, please consider the following:


**Methods**


-Are the objectives of the study clearly articulated with a clear testable hypothesis stated?

-Is the study design appropriate to address the stated objectives?

-Is the population clearly described and appropriate for the hypothesis being tested?

-Is the sample size sufficient to ensure adequate power to address the hypothesis being tested?

-Were correct statistical analysis used to support conclusions?

-Are there concerns about ethical or regulatory requirements being met?

Reviewer #1: -Yes
-Yes,the use of existing databases is appropriate for a study of this nature.
-Yes
-The authors have utilized the maximum available data from the selected European countries, which is sufficient for the predictive modeling proposed.
-Yes
-While the statistical framework is robust and the use of R packages is well-documented in the Supporting Information (SI), the complexity and sheer number of predictive models make it difficult to follow the main narrative.
-Yes

- Thank you for your careful consideration and positive assessment. As mentioned above, we have included a flowchart in the Methods section (lines 168-173) to help clearly communicate our analyses and main narrative.

**Results**


-Does the analysis presented match the analysis plan?

-Are the results clearly and completely presented?

-Are the figures (Tables, Images) of sufficient quality for clarity?

Reviewer #1: -Yes
-The results are comprehensive, but the presentation is overwhelming.
-No, the coefficient plots are too large and difficult to read. The maps for France and Spain lack the necessary resolution to be informative. It is unclear if this is a compression issue within the journal's PDF generator or the original file quality, but in their current state, they do not support the results effectively.

- Thank you again for your constructive feedback. With the revised manuscript, we have uploaded all figures according to journal requirements (resolution, file type, size, etc.). We now separately plot the risk factors per country to improve the readability. We hope these files now easily readable, effectively supporting the results.

**Conclusions**


-Are the conclusions supported by the data presented?

-Are the limitations of analysis clearly described?

-Do the authors discuss how these data can be helpful to advance our understanding of the topic under study?

-Is public health relevance addressed?

Reviewer #1: -The data supports the general conclusions, the link between the complex statistical models and the biological reality of leishmaniasis transmission could be more explicitly stated.
-Partially
-Yes
-Yes

- Thank you for your constructive feedback. In the Discussion, we now elaborate in greater detail on the link between model outcomes and the biological transmission cycle (lines 404-412).

**Editorial and Data Presentation Modifications?**


Use this section for editorial suggestions as well as relatively minor modifications of existing data that would enhance clarity. If the only modifications needed are minor and/or editorial, you may wish to recommend “Minor Revision” or “Accept”.

Reviewer #1: (No Response)

**Summary and General Comments**


Use this section to provide overall comments, discuss strengths/weaknesses of the study, novelty, significance, general execution and scholarship. You may also include additional comments for the author, including concerns about dual publication, research ethics, or publication ethics. If requesting major revision, please articulate the new experiments that are needed.

Reviewer #1: The manuscript presents a very detailed and technically robust statistical framework (as seen in the SI). However, the methodology is difficult to follow due to the sheer number of predictive models and formulas. I strongly recommend including a flowchart to guide the reader through the different stages of the analysis (from SIR calculations to the Bayesian INLA models).

Regarding the Discussion, there are two points that require further elaboration:

The authors mention that homeless populations are at higher risk due to exposure and malnutrition, yet they report no correlation with poverty indices (S2). This contradiction should be explored: is it a limitation of the databases used to represent the most vulnerable groups?

The findings in France regarding the lack of geographic expansion under future climate scenarios contradict previous literature. The authors state that socioeconomic factors outweighed climate, but this counterintuitive result needs a more thorough justification.

Finally, the visual presentation must be improved. The coefficient plots are 'noisy' and the maps lack the necessary resolution for a journal of this caliber.

- Thank you for your positive evaluation and constructive feedback. As noted in the responses to Reviewer 1, we have now added a flowchart in the Methods section (lines 168-173) to guide readers through our analysis. We have also added in the Discussion further elaborations regarding the apparent contradictions involving 1) homeless population and poverty indices (lines 376-379) and 2) climate and socioeconomic factors (lines 404-412). Finally, we have submitted with the revised manuscript all figures according to the journal’s technical requirements.
