# Supplementary material for: Socioeconomic risk factors and human immunosuppressive conditions are major drivers of human leishmaniasis in southern Europe
Source: PLoS Negl Trop Dis. 2026 Oct 5;20(10):e0014787. doi: 10.1371/journal.pntd.0014787 (PMC13649131; doi:10.1371/journal.pntd.0014787)
Supplement: Supplementary file 3 [file pntd.0014787.s006.docx]

Response to Reviewers

Dear editors and dear reviewer,

Thank you for the time you invested in reviewing and providing constructive feedback on our submitted manuscript. We incorporated all comments in a revised manuscript, and we outline the changes in detail below (starting with 🡪). We hope that the revised manuscript will now be suitable for publication in the journal. Thank you again for your consideration.

Best regards,

Danyang Wang

On behalf of all co-authors

**Reviewers' Comments:**

Reviewer's Responses to Questions

**Key Review Criteria Required for Acceptance?**


As you describe the new analyses required for acceptance, please consider the following:


**Methods**


-Are the objectives of the study clearly articulated with a clear testable hypothesis stated?

-Is the study design appropriate to address the stated objectives?

-Is the population clearly described and appropriate for the hypothesis being tested?

-Is the sample size sufficient to ensure adequate power to address the hypothesis being tested?

-Were correct statistical analysis used to support conclusions?

-Are there concerns about ethical or regulatory requirements being met?

Reviewer #1: -Yes, they are.
-Yes, It is.
-Yes, It is.
-Yes, It is.
-Yes, They were correct.
-Not, It aren't.

**Results**


-Does the analysis presented match the analysis plan?

-Are the results clearly and completely presented?

-Are the figures (Tables, Images) of sufficient quality for clarity?

Reviewer #1: -Yes, It does.
-Yes, they are.
-The inclusion of the methodology flowchart is appreciated and clarifies the analytical pipeline. However, its visual execution should be refined.

- Thank you for your careful evaluation. We now revised the methodology flowchart (line 168) with improved readability. The cylinder shapes are replaced by rectangle boxes.

**Conclusions**


-Are the conclusions supported by the data presented?

-Are the limitations of analysis clearly described?

-Do the authors discuss how these data can be helpful to advance our understanding of the topic under study?

-Is public health relevance addressed?

Reviewer #1: Yes, they are.
-Yes, they are.
-Yes, they do.
-Yes, It is.

**Editorial and Data Presentation Modifications?**


Use this section for editorial suggestions as well as relatively minor modifications of existing data that would enhance clarity. If the only modifications needed are minor and/or editorial, you may wish to recommend “Minor Revision” or “Accept”.

Reviewer #1: Minor Revision

**Summary and General Comments**


Use this section to provide overall comments, discuss strengths/weaknesses of the study, novelty, significance, general execution and scholarship. You may also include additional comments for the author, including concerns about dual publication, research ethics, or publication ethics. If requesting major revision, please articulate the new experiments that are needed.

Reviewer #1: The authors have done an excellent job addressing the previous comments. The methodological workflow is much clearer, the separation of coefficient plots by individual country (Figures 4 - 8) substantially improves readability, and the conceptual framing added to the Discussion (lines 376–379 and 404–415) satisfactorily addresses the previous concerns regarding poverty indices and climate projections in France.
Before final acceptance, a few minor technical and formatting issues in the figures should be addressed:
Figure 1:
The cylinder symbols representing datasets in the pre-processing column are visually compressed, causing the internal lines to overlap with the text. I suggest, replacing these cylinders with standard rectangular boxes or simplifying the icon outlines would greatly enhance readability.
Figure 2:
There are noticeable typographical errors in the province labels inside the maps: in panel (a), "Madri" should be "Madrid"; in panel (b), "Teleda" and "Cordopjaen" are misspelled/overlapping.

- Thank you for your positive evaluation and constructive feedback. We have now refined the visual presentation of figure 1 in the Methods section (line 168) by replacing cylinder shapes by rectangle boxes. We also corrected the typing and overlapping issues in figure 2 (line 233).
